# Development and Characterization of Econazole Topical Gel

**DOI:** 10.3390/gels9120929

**Published:** 2023-11-25

**Authors:** Mohammad F. Bayan, Balakumar Chandrasekaran, Mohammad H. Alyami

**Affiliations:** 1 Faculty of Pharmacy, Philadelphia University, P.O. Box 1, Amman 19392, Jordan; balakumar@philadelphia.edu.jo; 2 Department of Pharmaceutics, College of Pharmacy, Najran University, Najran 66462, Saudi Arabia; mhalmansour@nu.edu.sa

**Keywords:** econazole, cutaneous candidiasis, Carbopol^®^ 940, topical gel, sustainability

## Abstract

The purpose of this work was to develop a novel topical formulation of econazole nitrate based on gel that can be easily scaled up in one pot for the potential treatment of fungal and yeast infections. Econazole nitrate, a topical antifungal, is used to treat tinea versicolor, tinea pedis, and tinea cruris. Compared to applying cream or ointment, topical gels offer numerous advantages, one of which is that the drug is released more quickly to the intended site of action. A viscous mixture of propylene glycol, Capmul^®^ MCM C8, methyl and propyl paraben, and econazole nitrate were mixed together before being formulated into the optimized Carbopol^®^ gel bases. The gel’s color, appearance, and homogeneity were assessed visually. For every formulation, the drug content, pH, viscosity, spreadability, and gel strength were characterized. The cup plate diffusion method was used to evaluate the anti-fungal activity of the prepared formulations. To assess the behavior of the developed system, studies on in vitro release and mechanism were conducted. The manufactured formulations were transparent, pale yellow, and exhibited excellent homogeneity. The pH of each formulation was roughly 6.0, making them suitable for topical use. The concentration of Carbopol^®^ 940 resulted in a significant increase in viscosity and gel strength but a significant decrease in spreadability. It was demonstrated that the prepared formulations inhibited the growth of *Candida albicans* and *Aspergillus fumigatus*. In contrast, the standard blank gel showed no signs of antifungal action. By increasing the concentration of Carbopol^®^ 940, the in vitro release profile of econazole nitrate significantly decreased. Following the Korsmeyer–Peppas model fitting, all formulations exhibited n values greater than 0.5 and less than 1, indicating that diffusion and gel swelling control econazole nitrate release.

## 1. Introduction

A wound is defined as a disruption of the anatomical or functional continuity of living tissue. The majority of wound healing research is focused on the skin, which is the body’s most susceptible organ and is affected by environmental interactions. New topical formulations that are physically, chemically, and biologically stable have drawn increasing attention from scientists in recent decades. Inorganic and organic molecules are encased and interpenetrated by a liquid in the gel formulation. The purpose of these pharmaceutical formulations is to minimize adverse effects while achieving targeted drug delivery. The gel-based system should be washable, non-staining, and stable at room temperature. Being simple to administer and transfer, they have the major benefit of avoiding the hepatic first pass. The use of topical drug delivery systems is growing, and many medications have been effectively administered this way for both systemic and local action. Topical antifungal therapy is thought to be the first line of treatment for superficial fungal infections of the skin, which are very common [1]. Compounds with antifungal activity against yeasts, molds, or both can be found naturally or artificially produced. Since both fungi and mammalian cells are eukaryotes, antifungal drugs that prevent the synthesis of proteins, RNA, and DNA may be harmful to mammalian cells [2]. There are numerous topical antifungals with low systemic side effects and high efficacy available. The well-known azole antifungals miconazole, clotrimazole, and ketoconazole have been used widely to treat dermatophytoses. Whereas ketoconazole works well for treating seborrheic dermatitis, miconazole and clotrimazole are effective in treating cutaneous candidiasis [3]. The two most recent azoles to come to light are efinaconazole and luliconazole. Efinaconazole has been approved for the treatment of onychomycosis, along with tavaborole, a novel agent with a unique mechanism of action. Luliconazole has been approved for the treatment of tinea corporis, tinea cruris, and tinea pedis. A broad-spectrum antifungal drug called econazole nitrate is used to treat skin infections brought on by different pathogenic dermatophyte species. It is applied to treat candidiasis [4]. Fungal infections typically affect the mucous membranes, skin, hair, and nails, but they can also infect the lungs or other sections of your body. Some individuals, typically those with compromised immune systems, develop potentially fatal systemic candidiasis when Candida invades deeper tissues in addition to the blood. One of the most frequent species of Aspergillus that sickens immunocompromised people is *Aspergillus fumigatus*. Although fungus infections of the skin, hair, and nails are normally not dangerous, they can take some time to fully heal even with treatment [5]. Nowadays, a range of antifungal creams are used to treat various mycotic infections and dermatological conditions. Nevertheless, it has been demonstrated that a large number of these fungal infections are resistant to treatment and control measures. Furthermore, patients receiving topical treatment may experience local reactions such as burning, erythema, stinging, pruritic rash, and tenderness [6]. Additional issues with the creams include failed stability tests, chemical stability, or physical emulsion separation brought on by the imidazole salt’s salting effect when used at concentrations of at least 1%. Topical administration of gels at pathological locations offers major advantages over cream and ointment in terms of faster absorption and direct drug release from medicine. The use of single-phase gel is common in skin care products [7]. Econazole irritates the gastrointestinal tract if taken orally. Topical gel can maximize drug concentration at the infected site of action and avoid the first-pass effect [8]. An ideal topical formulation should spread evenly and leave no residue behind. Carbopol^®^ 940 is frequently used in gel-based formulations due to its exceptional viscosity building properties (even at low concentrations) and resistance to microbial development [9]. Econazole nitrate is commonly taken once or twice a day for 14 days, in the morning and evening, and up to 42 days in serious infections. Since econazole nitrate is a BCS class 2 drug with low solubility and high permeability, its solubility must be improved. The most difficult issue facing the pharmaceutical industry is solubility and permeability, which affects more than 65% of newly discovered medicinal agents as they fall into BCS classes 2 and 4 [10]. The poor solubility of econazole nitrate can be solved by incorporating it in a topical gel using propylene glycol as a plasticizer, Capmul^®^ MCM C8 as an emollient and solubilizer, methyl and propyl paraben as preservatives, and Carbopol^®^ 940 as a gelling agent. The goal of this work was to develop a novel gel-based topical formulation of econazole nitrate (Figure S1) that could be readily scaled up in a single pot to treat possible yeast and fungal infections. The physicochemical properties of the prepared formulations were evaluated, and in vitro release and mechanism studies were carried out to evaluate the behavior of the developed system.

## 2. Results and Discussion

### 2.1. Formulation of Econazole Topical Gel

Water, alcohol, or another solvent (like propylene glycol) and a hydrophilic polymer are the two main ingredients of a gel. In the vast majority of water-based gels, the hydrophilic polymer acts as a gelling agent using water. The viscosity of the produced gels is mainly related to the amount of gelling agent used. Those that yield a firmer gel, on the other hand, usually cause it to become more fragile and brittle [11]. In the process of creating a gel, the two most important ingredients are the solubilizer and gelling agent. The excipients are used to determine whether a gel is clear, translucent, or opaque [12]. A difficult part of the formulation process is selecting the right gelling agent and keeping a drug in its solubilized state. The desired physical characteristics of the gel were achieved by optimizing different concentrations of Carbopol^®^ 940: 0.75–2%. Topical antifungal econazole nitrate is used to treat cutaneous candidiasis, tinea versicolor, tinea pedis, and tinea cruris. Topical gels have many benefits over cream or ointment application, including a quicker release of medication to the intended site of action [13]. A viscous mixture of propylene glycol (plasticizer), Capmul^®^ MCM C8 (emollient and solubilizer), methyl and propyl paraben (preservatives) was used to dissolve econazole nitrate. This mixture was then added to the optimized Carbopol^®^ gel bases. Table 1 contains the formulations for the econazole nitrate topical gel that were designed.

### 2.2. Physical Characterization of the Formulated Gel

The formulated gel was checked visually for color, appearance, and homogeneity, and the results are listed in Table 2. Physical examination of the prepared formulations revealed that they were transparent, pale yellow, and had excellent homogeneity (Figure S2).

### 2.3. pH Determination

The pH measures the degree of acidity or alkalinity. It is important to the pharmaceutical industry because certain drugs can lose their potency or degrade in acidic or alkaline environments; some drugs can only dissolve partially in acidic or alkaline media, which can affect their bioavailability and therapeutic efficacy, and the pH of biological fluids, such as blood and urine, can change the rate and degree of drug release. This advocates the importance of pH in the development, manufacturing, and administration of pharmaceutical products. A pH between 4.2 and 6.5 is considered suitable for topical preparations on the skin. Scaly skin is a result of overly alkaline gels. Conversely, the skin will become irritated if the pH is excessively acidic. The pH was approximately 6.0 for all formulations (Table 3). This suggests that it is skin-compatible and appropriate for topical use [14].

### 2.4. Determination of Viscosity

**Figure 1:**
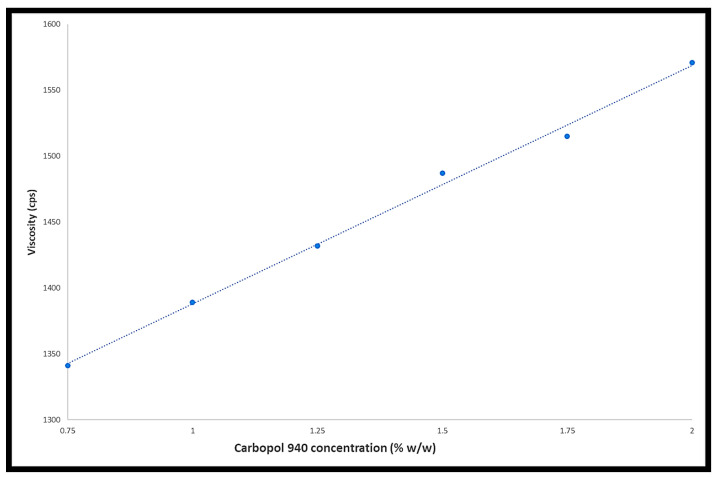
The effect of Carbopol^®^ 940 concentration on viscosity.

Gels’ rheological properties vary, and they exhibit reversible deformation instead of flowing at low shear stresses, which is comparable to what happens to elastic materials. When the yield value of shear stress is exceeded, they flow like liquids. Generally speaking, the viscosity of gel compositions indicates their consistency. Shear thinning, also known as non-Newtonian flow, illustrates how gel viscosity decreases as the shear rate increases, which is preferred as gels work well in high shear conditions because of their low flow resistance [15]. Drug diffusion from a gel is influenced by the rheological property, which also aids in consistency determination. Improved flow and spreadability allow for more precise application over the skin while preserving the benefits of more aesthetically pleasing properties at a viscosity below 15,000 cps. This low viscosity also indicates that the gel will behave in a viscoelastic manner when stress is applied, which will facilitate flow from the container to the applying area and suction back to the container when the stress releases. Table 3 and Figure 1 present the viscosity measurement results. The low viscosity of all the formulations is evident, indicating a promising application for topical administration. It can also be observed that viscosity increased from F1 to F6, which can be attributed to the increased concentration of Carbopol^®^ 940 [16].

### 2.5. Spreadability

**Figure 2:**
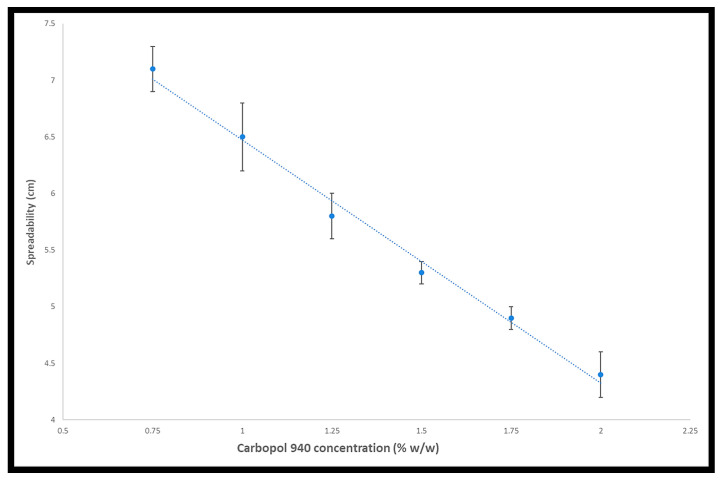
The effect of Carbopol^®^ 940 concentration on spreadability.

Since the spreadability of the gel helps with the uniform application of the gel to the skin, manufactured gels must have good spreadability and meet the ideal quality in topical application. This is also considered to be a crucial component of patient compliance with the treatment. How easily a gel spreads when applied topically is referred to as its spreadability [17]. Table 3 and Figure 2 present the spreadability measurements of all formulations. It can be observed that spreadability reduced from F1 to F6. This suggests that spreadability is negatively impacted by increasing the concentration of Carbopol^®^ 940.

### 2.6. Gel Strength

**Figure 3:**
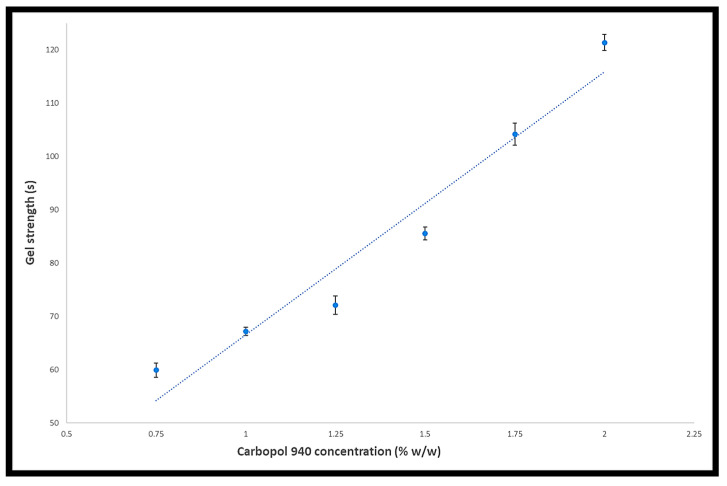
The effect of Carbopol^®^ 940 concentration on gel strength.

Products like gels, capsules, and contact lenses are developed based on their strength, flexibility, and rupture force. Gel strength is a measure of a colloidal dispersion’s capacity to form and sustain a gel form. Strong gels will withstand far greater pressure than weak gels before being flushed from the site of administration, and it will affect the spreadability and drug’s release. This makes gel strength crucial. Table 3 and Figure 3 display the gel strengths of the developed formulations. Increasing the concentration of Carbopol^®^ 940 increased the gel strength [18].

### 2.7. Anti-Fungal Activity

The cup plate method involves adding a test organism to the nutrient agar plate to a depth of 5 mm, allowing it to solidify. The plate is then divided into four equal parts, and in each part, a cavity is created using a sterile cork borer. One of the four created cavities is filled with a standard and the other three with the medicinal agent and incubated for 24 h at 37 °C prior measuring the inhibited zones. This approach is accessible for the routine assay of biological materials, and it is straightforward and accurate. The zone of inhibition (measured in millimeters), which represents the microorganism’s susceptibility to the medicinal agents, was measured to assess the anti-fungal activity of the developed gel formulations. Aspergillus fumigatus and Candida albicans were shown to be inhibited in growth by the gel formulations F1–F6 (Table 4); however, no antifungal activity was observed in the standard blank gel (which was not loaded with econazole nitrate).

### 2.8. Drug Content

A vehicle and an active component are both present in topical preparations. Water, oil, alcohol, or propylene glycol combined with other ingredients, such as emulsifiers, absorption enhancers, and preservatives, are typically found in the vehicle. This necessitates the need to determine the drug content of the topical formulation to ensure it contains the proper amount. According to Table 5, the drug content findings of the formulations loaded with econazole nitrate fell between 90.0% and 110.0%, which falls within the USP specification [19]. The theoretical drug content is 1 mg, and the actual drug content ranged from 0.96 to 1.08 mg, which is equivalent to 96–108%.

### 2.9. In Vitro Release Studies

**Figure 4:**
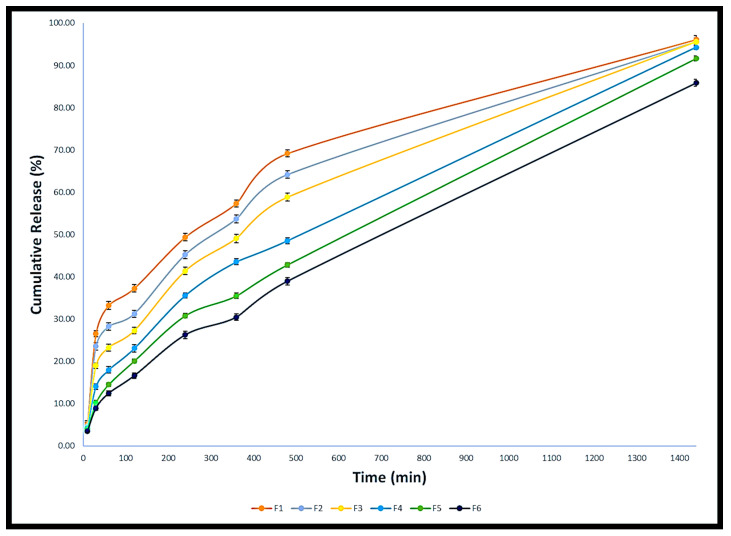
Econzaole nitrate in vitro release profiles.

**Figure 5:**
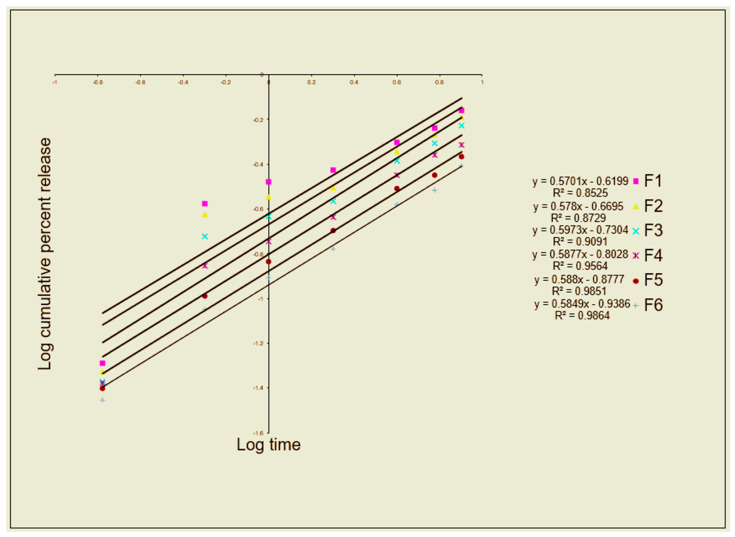
Korsmeyer–Peppas model release kinetics of econazole nitrate formulations.

To help deliver the medication to the sites of action, controlled drug delivery systems based on topical preparations have been developed. This can improve patient compliance as well as the medication’s safety and effectiveness [20]. Econazole nitrate’s release profile from the created gel formulations was examined (Figure 4). The values of the release rate constants (k), R^2^, and n, after the Korsmeyer–Peppas model, were applied, as shown in Table 6. Consistent with the studies on viscosity, spreadability, and gel strength, the release profile of econazole nitrate significantly decreased as the concentration of Carbopol^®^ 940 increased. To investigate the drug release mechanism and the release rate constant (k), the first 60% of the release data were fitted to the Korsmeyer–Peppas model (Table 6 and Figure 5). The drug release is studied and explained by the Korsmeyer–Peppas model when it occurs from a gel-based dosage form or when the release follows several kinetics mechanisms. Table 6 shows that all formulations had n values more than 0.5 and less than 1, which suggests an anomalous drug release mechanism. This suggests that the release of econazole nitrate is regulated by diffusion and gel swelling [21].

### 2.10. Stability Studies

The process of monitoring products for predefined periods of time, particularly environmental settings, in order to ascertain whether there has been any change in quality, is known as stability testing. After 180 days of storage at room temperature, it was observed that the physicochemical properties—color, appearance, homogeneity, pH, viscosity, spreadability, gel strength, and drug content—were maintained (Tables S1–S6), demonstrating the stability of the created formulations [22]. 

## 3. Conclusions

Due to their structural flexibility, high stability, and ease of manufacture, topical gel compositions have become indispensable as drug delivery vehicles. The benefits of topical administration over traditional techniques are numerous, including noninvasive delivery, avoiding first-pass metabolism, and increased patient compliance. This study looked into the development of a novel one-pot gel based topical formulation of econazole nitrate for the potential treatment of yeast and fungal infections. Six formulations were prepared with success, and each formulation had a satisfactory drug content. The manufactured formulations were transparent, pale yellow, and exhibited excellent homogeneity. All formulations had a pH of about 6.0, which qualifies them for topical application. Viscosity and gel strength increased significantly with the Carbopol^®^ 940 concentration, but the spreadability decreased significantly. The developed formulations were shown to suppress the growth of *Aspergillus fumigatus* and *Candida albicans*. In contrast, no antifungal activity was detected in the standard blank gel, which was not loaded with econazole nitrate. The in vitro release profile of econazole nitrate decreased significantly by increasing the Carbopol^®^ 940 concentration. After fitting to the Korsmeyer–Peppas model, all formulations had n values more than 0.5 and less than 1, which suggests that the release of econazole nitrate is regulated by diffusion and gel swelling. The outcomes of this study have confirmed the developed formulation’s potential application as a promising approach to treat fungal and yeast infections, one that can be easily scaled up in a single pot.

## 4. Materials and Methods

### 4.1. Materials

The following supplies were bought from Sigma-Aldrich, Merck Life Science UK Limited, Glasgow, Scotland: econazole nitrate, Carbopol^®^ 940, propylene glycol, methylparaben, propylparaben, disodium hydrogen phosphate dodecahydrate, triethanolamine, potassium chloride, sodium chloride, potassium dihydrogen phosphate, sodium dodecyl sulphate, methanol, and sodium hydroxide. The supplier of Capmul^®^ MCM C8 was ABITEC corporation, Surrey, England. Aspergillus fumigatus (ATCC 1022) and Candida albicans (ATCC 18804) were purchased from ATCC, Manassas, VA, USA. All of the experiments used water of HPLC quality. The purchased materials were used exactly as they were supplied.

### 4.2. Methods

#### 4.2.1. Formulation of Econazole Topical Gel

The gelling agent Carbopol^®^ 940 was added to a sufficient amount of water at different concentrations (Table 1) and agitated for two hours at 100 rpm. The resultant mixture was neutralized with triethanolamine. Additionally, econazole nitrate was dissolved in a viscous mixture of propylene glycol (plasticizer), Capmul^®^ MCM C8 (emollient and solubilizer) as well as methyl and propyl paraben (preservatives). After that, this mixture was added to the gel base and thoroughly mixed with the addition of distilled water to bring the gel’s ultimate weight to 100 g. Following two hours of shaking at 500 rpm to eliminate any air bubbles, the aforementioned combination was left to be evaluated for stability and consistency for a full day at room temperature [7].

#### 4.2.2. Physical Characterization of the Produced Gel

After being packed into the container, the formulated gel’s physical characteristics, color, and homogeneity were visually assessed.

#### 4.2.3. Determination of pH

The pH scale indicates how acidic or alkaline a solution is. It is significant to the pharmaceutical industry because different drugs react differently to acidic or alkaline environments, varying in potency or degradation; some drugs only partially dissolve in these conditions, affecting their bioavailability and therapeutic efficacy. Moreover, biological fluid pH, such as blood and urine, can alter the rate and extent of drug release. A pH in the range from 4.2 to 6.5 is thought to be appropriate for topical skin treatments. Gels that are excessively alkaline cause scaly skin. On the other hand, an overly acidic pH will irritate the skin. One hundred milliliters of purified water were combined with about two grams of the prepared gel formulation. Using a digital pHmeter (Sartorius- PB-10 Basic Bench Top pH Meter, Sartorius group, Göttingen, Germany) at room temperature, the pH of each formulation was measured three times to produce triplicate readings.

#### 4.2.4. Viscosity Determination

The viscosity of the prepared formulations was measured using a Brookfield RVT-cup-and-bob rotational viscometer (Middleborough, MA, USA) with spindle No. 62 rotated at 25 rpm at 25 °C. The viscosity of each formulation was measured three times to produce triplicate readings.

#### 4.2.5. Spreadability

The spreadability of formulations was assessed to see how much of the skin they would cover when applied. A 500 mg gel was applied over a 3 cm diameter circle that had been drawn in the center of the glass plate. After that, this plate was covered with a second plate weighing 0.5 kg. The circle’s diameter was then measured after six min. The measurement was repeated three times [23].

#### 4.2.6. Gel Strength

A 30 mm stainless-steel ball was inserted into the center of 40 g of the gel, which was then permitted to pierce through the gel, into a 50 mL beaker. For every formulation, the duration required for the ball to pierce the gel five centimeters down was recorded [18]. The measurement was repeated three times for each formulation.

#### 4.2.7. Anti-Fungal Activity

The produced econazole loaded formulations were assessed for their anti-fungal activity using the cup plate diffusion method as previously described [7]. Standard cultures of Aspergillus fumigatus ATCC 1022 and Candida albicans ATCC 18804 were used, along with a blank gel (an unloaded formulation based on F6) as a standard. To cut the agar medium, a sterile cork borer no. 3 (7.5 mm) was utilized. The plates were incubated for 24 h at 37 °C prior determining the zone of inhibition. The measurement was repeated three times.

#### 4.2.8. Drug Content

**Figure 6:**
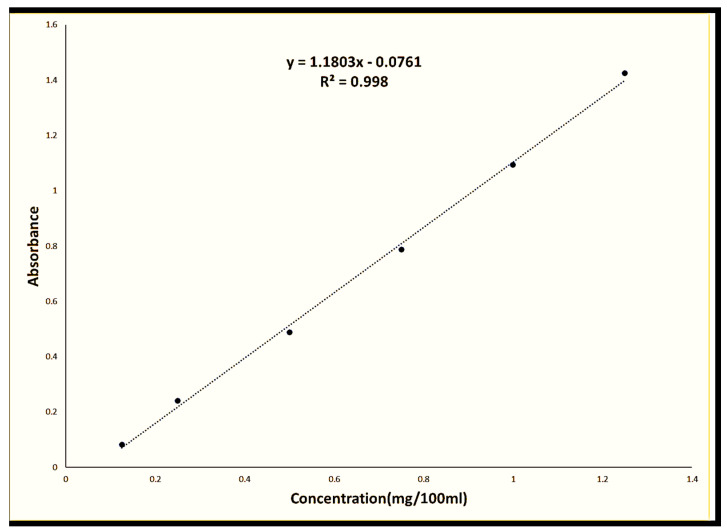
Calibration curve of econzaole nitrate.

The drug content of the prepared formulations was determined by placing 100 mg of the gel, which is equivalent to 1 mg of econazole nitrate, in 100 mL of methanol inside a shaking bath (Biobase thermostatic shaking bath-SWB-A, Jinan, China), operating at 37 °C and 100 cycles per min. After that, the samples were filtered through a 0.45-micron filter membrane, and a UV/VIS spectrophotometer (Spectroscan 80 D, Nicosia, Cyprus) was used to determine their absorbance at 271 nm [19]. The content was calculated using the equation obtained from the calibration curve (Figure 6). The measurement was repeated three times for each formulation.

#### 4.2.9. In Vitro Release Studies

The in vitro release of econazole nitrate was investigated in 25% v/v methanolic PBS (pH 7.4) at 37 °C using a Franz diffusion cell assisted by a cellophane membrane. For every formulation, triplicates were carried out. Five milliliters of the sample and five milliliters of fresh media were switched out at predetermined intervals. Using a UV/VIS spectrophotometer (Spectroscan 80 D, Nicosia, Cyprus), the absorbance of the withdrawn samples was determined at 271 nm [21]. For econazole nitrate in methanol, a fully validated calibration curve was created (Figure 6). The Korsemeyer–Peppas model was used to fit the first 60% of the data in order to investigate the rate and mechanism of drug release. This model serves as a straightforward relationship to determine the drug release mechanism. The entire set of data was statistically evaluated using a two-way analysis of variance test, and the data (n = 3, *p* 0.05) were statistically evaluated using Tukey’s multiple comparison test.

#### 4.2.10. Stability Studies

Stability studies are vital when assessing a product’s shelf life. The product is subjected to specific conditions for the required time in stability chambers. Key data such as concentration, active component, appearance, color change, and mold or bacteria development are recorded at predefined intervals. A long-term stability study is carried out that, in accordance with recommendations, should last at least six months for pharmaceutical products containing active medicinal components currently in use. The climatic circumstances in which the product is meant to be stored dictated the study’s conditions, which were 25 °C ± 2 °C/60% RH ± 5% RH. For 180 days, the prepared formulations were kept at room temperature in a closed glass container. The physicochemical characteristics of the samples—color, appearance, homogeneity, pH, viscosity, spreadability, gel strength, and drug content—were evaluated and analyzed according to the published methodology [22].

## Figures and Tables

**Table 1:** The ingredients of the prepared formulations.

Ingredients	F1	F2	F3	F4	F5	F6
Econazole nitrate	1.00%	1.00%	1.00%	1.00%	1.00%	1.00%
Capmul^®^ MCM C8	5.00%	5.00%	5.00%	5.00%	5.00%	5.00%
Carbopol^®^ 940	0.75%	1.00%	1.25%	1.50%	1.75%	2.00%
Propylene glycol	20.00%	20.00%	20.00%	20.00%	20.00%	20.00%
Methyl Paraben	0.15%	0.15%	0.15%	0.15%	0.15%	0.15%
Propyl paraben	0.30%	0.30%	0.30%	0.30%	0.30%	0.30%
Triethanolamine	2.00%	2.00%	2.00%	2.00%	2.00%	2.00%
Water (q. s)	100%	100%	100%	100%	100%	100%

**Table 2:** Physical characterization of the prepared formulations.

Characteristics	F1	F2	F3	F4	F5	F6
Physical appearance	Transparent	Transparent	Transparent	Transparent	Transparent	Transparent
Color	Pale yellow	Pale yellow	Pale yellow	Pale yellow	Pale yellow	Pale yellow
Homogeneity	No aggregates	No aggregates	No aggregates	No aggregates	No aggregates	No aggregates

**Table 3:** The pH, viscosity, spreadability, and gel strength measurements.

Formulation Code	pH	Viscosity (cps)	Spreadability (cm)	Gel Strength (s)
F1	6.1 ± 0.1	1341 ± 0.6	7.1 ± 0.2	59.9 ± 1.3
F2	6.0 ± 0.1	1389 ± 0.9	6.5 ± 0.3	67.2 ± 0.8
F3	6.1 ± 0.1	1432 ± 0.8	5.8 ± 0.2	72.1 ± 1.7
F4	5.9 ± 0.2	1487 ± 0.5	5.3 ± 0.2	85.6 ± 1.2
F5	6.2 ± 0.1	1515 ± 0.6	4.9 ± 0.1	104.2 ± 2.1
F6	5.9 ± 0.1	1571 ± 0.8	4.4 ± 0.2	121.4 ± 1.5

**Table 4:** The zone of inhibition (mm) against *Aspergillus fumigatus* and *Candida albicans*.

Sample	*Aspergillus fumigatus*	*Candida albicans*
Blank gel	-	-
F1	14.15 ± 1.16	16.47 ± 1.49
F2	14.54 ± 1.34	16.22 ± 1.72
F3	15.21 ± 1.02	16.63 ± 1.53
F4	14.89 ± 1.44	16.45 ± 1.30
F5	14.74 ± 1.68	16.88 ± 1.37
F6	15.36 ± 1.67	16.21 ± 1.81

**Table 5:** Drug content of the prepared formulations.

Formulation	Drug Content (mg)
F1	0.99 ± 0.056
F2	0.96 ± 0.030
F3	0.98 ± 0.034
F4	1.08 ± 0.035
F5	1.02 ± 0.085
F6	1.01 ± 0.081

**Table 6:** Fitting to the Korsmeyer–Peppas model.

Formulation	Korsmeyer–Peppas R^2^	n Value	K_m_
F1	0.853	0.570	0.240
F2	0.873	0.578	0.214
F3	0.909	0.597	0.186
F4	0.956	0.588	0.157
F5	0.985	0.588	0.133
F6	0.986	0.585	0.115

## Data Availability

All data are available in the manuscript.

## Supplementary Materials

The following supporting information can be downloaded at: https://www.mdpi.com/article/10.3390/gels9120929/s1, Figure S1: Chemical Structure of Econazole Nitrate; Figure S2: A picture of the topical econazole gel formulation; Table S1: Stability of F1; Table S2: Stability of F2; Table S3: Stability of F3; Table S4: Stability of F4; Table S5: Stability of F5; Table S6: Stability of F6.
