# Supplementary material for: Development and Characterization of Econazole Topical Gel
Source: Gels. 2023 Nov 25;9(12):929. doi: 10.3390/gels9120929 (PMC10743284; doi:10.3390/gels9120929)
Supplement: Supplementary file 1 [file gels-09-00929-s001.zip › gels-2733443-supplementary.pdf]

Supplementary Materials: Development and Characterization of Econazole Topical Gel

Mohammad F. Bayan [1,*], Balakumar Chandrasekaran [1] and Mohammad H. Alyami [2]

[1] Faculty of Pharmacy, Philadelphia University, P.O. Box 1, Amman 19392, Jordan; balakumar@philadelphia.edu.jo
[2] Department of Pharmaceutics, College of Pharmacy, Najran University, Najran 66462, Saudi Arabia;
mhalmansour@nu.edu.sa
* Correspondence: mbayan@philadelphia.edu.jo

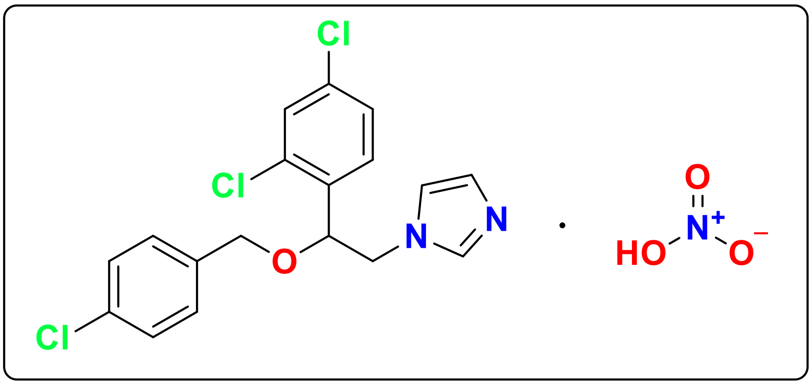

Figure S1. Chemical Structure of Econazole Nitrate

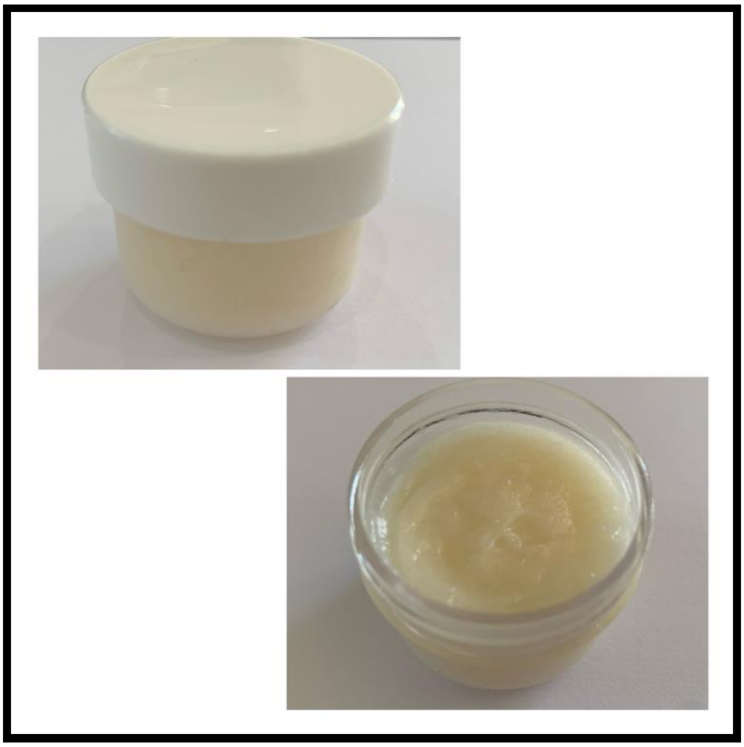

Figure S2. A picture of the topical econazole gel formulation

Table S1. Stability of F1

Time (days)	pH	Viscosity (cps)	Spreadability (cm)	Gel strength (s)
0	6.1 ± 0.1	1341 ± 0.6	7.1 ± 0.2	59.9 ± 1.3
30	6.2 ± 0.2	1342 ± 0.8	7.0 ± 0.4	58.7 ± 1.1
90	6.0 ± 0.2	1341 ± 0.7	7.1 ± 0.1	58.2 ± 1.6
180	6.1 ± 0.2	1342 ± 0.5	7.1 ± 0.2	59.5 ± 1.2

Table S2. Stability of F2

Time (days)	pH	Viscosity (cps)	Spreadability (cm)	Gel strength (s)
0	6.0 ± 0.1	1389 ± 0.9	6.5 ± 0.3	67.2 ± 0.8
30	5.9 ± 0.2	1390 ± 1.0	6.3 ± 0.2	66.8 ± 0.9
90	6.0 ± 0.1	1389 ± 0.7	6.4 ± 0.3	68.4 ± 1.5
180	6.0 ± 0.2	1389 ± 0.8	6.3 ± 0.2	66.5 ± 1.4

Table S3. Stability of F3

Time (days)	pH	Viscosity (cps)	Spreadability (cm)	Gel strength (s)
0	6.1 ± 0.1	1432 ± 0.8	5.8 ± 0.2	72.1 ± 1.7
30	5.9 ± 0.3	1429 ± 1.5	5.8 ± 0.2	73.8 ± 2.0
90	5.9 ± 0.2	1431 ± 1.3	5.8 ± 0.1	70.6 ± 1.8
180	6.1 ± 0.2	1433 ± 1.4	5.7 ± 0.2	71.7 ± 1.1

Table S4. Stability of F4

Time (days)	pH	Viscosity (cps)	Spreadability (cm)	Gel strength (s)
0	5.9 ± 0.2	1487 ± 0.5	5.3 ± 0.2	85.6 ± 1.2
30	6.1 ± 0.2	1485 ± 0.8	5.1 ± 0.3	86.5 ± 1.4
90	6.0 ± 0.2	1486 ± 0.6	5.2 ± 0.3	85.9 ± 1.6
180	6.0 ± 0.2	1488 ± 1.1	5.2 ± 0.1	87.2 ± 1.7

Table S5. Stability of F5

Time (days)	pH	Viscosity (cps)	Spreadability (cm)	Gel strength (s)
0	6.2 ± 0.1	1515 ± 0.6	4.9 ± 0.1	104.2 ± 2.1
30	6.0 ± 0.1	1518 ± 1.9	4.8 ± 0.1	105.5 ± 2.6
90	6.0 ± 0.2	1517 ± 1.3	4.8 ± 0.1	106.4 ± 2.2
180	6.0 ± 0.1	1514 ± 1.2	4.9 ± 0.1	103.6 ± 1.9

Table S6. Stability of F6

Time (days)	pH	Viscosity (cps)	Spreadability (cm)	Gel strength (s)
0	5.9 ± 0.1	1571 ± 0.8	4.4 ± 0.2	121.4 ± 1.5
30	6.1 ± 0.1	1570 ± 0.9	4.3 ± 0.1	122.2 ± 1.8
90	6.0 ± 0.2	1572 ± 0.8	4.3 ± 0.2	121.6 ± 1.8
180	5.9 ± 0.2	1570 ± 1.1	4.3 ± 0.1	121.2 ± 1.7
